# Supplementary material for: Chromatin accessibility dynamics and single cell RNA-Seq reveal new regulators of regeneration in neural progenitors
Source: eLife. 2020 Apr 27;9:e52648. doi: 10.7554/eLife.52648 (PMC7250574; doi:10.7554/eLife.52648)
Supplement: Supplementary file 2. [file elife-52648-supp2.docx]

| **Supplementary Table 2: Key Resources Table** | | | | |
| --- | --- | --- | --- | --- |
| **Reagent type (species) or resource** | **Designation** | **Source or reference** | **Identifiers** | **Additional information** |
| Strain, strain background (*Xenopus tropicalis, female/male)* | Wild-type, Nigerian | NASCO | NASCO LM00822 |  |
| Strain, strain background (*Xenopus tropicalis, female/male)* | Strain, strain background (*Xenopus tropicalis, female/male)* | National *Xenopus* Resource, PMID: 12454928 | RRID:NXR_1.0021 |  |
| Antibody | Goat anti-Mouse IgG (H+L) Cross-Adsorbed Secondary Antibody, Alexa Fluor 488 | ThermoFisher Cat. A-11001 | RRID:AB_2534069 | 1:500 2hours at RT |
| Antibody | Goat anti-Mouse IgG (H+L) Cross-Adsorbed Secondary Antibody, Alexa Fluor 488 | ThermoFisher Cat. A-21422 | RRID:AB_2535844 | 1:500 2hours at RT |
| Antibody | Goat anti-Rabbit IgG (H+L) Cross- Adsorbed Secondary antibody, Alexa Fluor 594 | ThermoFisher Cat. A-11012 | RRID:AB_2534079 | 1:500 2hours at RT |
| Antibody | Anti-Neurofilament associated, mouse monoclonal | Developmental Studies Hybridoma Bank, Cat. 3A10 | RRID:AB_531874 | 1:50 overnight at 4C |
| Antibody | Anti-Doublecortin, rabit polyclonal | Cell Signaling Technology, 4604S | RRID:AB_561007 | 1:200 overnight at 4C |
| Antibody | Anti-Sox2, rabit polyclonal | Cell Signaling Technology, 2748S | RRID:AB_823640 | 1:100 overnight at 4C |
| Antibody | Anti-Histone H3 (tri methly K9, Phospho S10), mouse monoclonal | Abcam 14955 | RRID:AB_443110 | 1:1000 overnight at 4C |
| Commerical assay or kit | DAPI | Sigma D9542 | Sigma D9542 | 1:2000 10min at RT |
| Commerical assay or kit | Dextran (fluoro-tracer) | ThermoFisher D1817 | ThermoFisher D1817 | injected at 2mg/mL |
| Software, algorithm | Cellranger (v2.0.2) | 10x Genomics | RRID:SCR_017344 | <https://support.10xgenomics.com/single-cell-gene-expression/software/pipelines/latest/what-is-cell-ranger> |
| Software, algorithm | Seurat (V3.0) | PMID: 31178118 | RRID:SCR_016341 | <https://satijalab.org/seurat/> |
| Software, algorithm | Trim Galore! | Felix Krueger | RRID:SCR_011847 | <https://github.com/FelixKrueger/TrimGalore> |
| Software, algorithm | Bowtie2 (V2) | PMID: 22388286 ; PMID: 30020410 | RRID:SCR_005476 | <http://bowtie-bio.sourceforge.net/bowtie2/manual.shtml> |
| Software, algorithm | MACS2 (V2) | PMID: 18798982 | RRID:SCR_013291 | <https://github.com/taoliu/MACS> |
| Software, algorithm | GenomicRanges | PMID: 23950696 |  | <https://bioconductor.org/packages/release/bioc/html/GenomicRanges.html> |
| Software, algorithm | edgeR | PMID: 19910308; PMID: 22287627 | RRID:SCR_012802 | <https://bioconductor.org/packages/release/bioc/html/edgeR.html> |
| Software, algorithm | gprofiler2 (V2) | PMID: 31066453 | RRID:SCR_018190 | <https://cran.r-project.org/web/packages/gprofiler2/index.html> |
| Software, algorithm | ReviGO | PMID: 21789182 | RRID:SCR_005825 | <http://revigo.irb.hr/> |
| Software, algorithm | HOMER (V4.10) | PMID: 20513432 | RRID:SCR_010881 | <http://homer.ucsd.edu/homer/> |
| Sequence-based reagent | PMID: 24097267:Ad2.1; Nextera barcode 701 | [PMID: 24097267](https://www.ncbi.nlm.nih.gov/pubmed/24097267) | library primers | CAAGCAGAAGACGGCATACGAGATTCGCCTTAGTCTCGTGGGCTCGGAGATGT |
| Sequence-based reagent | PMID: 24097267:Ad2.2; Nextera barcode 702 | [PMID: 24097267](https://www.ncbi.nlm.nih.gov/pubmed/24097267) | library primers | CAAGCAGAAGACGGCATACGAGATCTAGTACGGTCTCGTGGGCTCGGAGATGT |
| Sequence-based reagent | PMID: 24097267:Ad2.3; Nextera barcode 703 | [PMID: 24097267](https://www.ncbi.nlm.nih.gov/pubmed/24097267) | library primers | CAAGCAGAAGACGGCATACGAGATTTCTGCCTGTCTCGTGGGCTCGGAGATGT |
| Sequence-based reagent | PMID: 24097267:Ad2.4; Nextera barcode 704 | [PMID: 24097267](https://www.ncbi.nlm.nih.gov/pubmed/24097267) | library primers | CAAGCAGAAGACGGCATACGAGATGCTCAGGAGTCTCGTGGGCTCGGAGATGT |
| Sequence-based reagent | PMID: 24097267:Ad2.5; Nextera barcode 705 | [PMID: 24097267](https://www.ncbi.nlm.nih.gov/pubmed/24097267) | library primers | CAAGCAGAAGACGGCATACGAGATAGGAGTCCGTCTCGTGGGCTCGGAGATGT |
| Sequence-based reagent | PMID: 24097267:Ad2.6; Nextera barcode 706 | [PMID: 24097267](https://www.ncbi.nlm.nih.gov/pubmed/24097267) | library primers | CAAGCAGAAGACGGCATACGAGATCATGCCTAGTCTCGTGGGCTCGGAGATGT |
| Sequence-based reagent | PMID: 24097267:Ad2.7; Nextera barcode 707 | [PMID: 24097267](https://www.ncbi.nlm.nih.gov/pubmed/24097267) | library primers | CAAGCAGAAGACGGCATACGAGATGTAGAGAGGTCTCGTGGGCTCGGAGATGT |
| Sequence-based reagent | PMID: 24097267:Ad2.8; Nextera barcode 708 | [PMID: 24097267](https://www.ncbi.nlm.nih.gov/pubmed/24097267) | library primers | CAAGCAGAAGACGGCATACGAGATCCTCTCTGGTCTCGTGGGCTCGGAGATGT |
| Sequence-based reagent | PMID: 24097267:Ad2.9; Nextera barcode 709 | [PMID: 24097267](https://www.ncbi.nlm.nih.gov/pubmed/24097267) | library primers | CAAGCAGAAGACGGCATACGAGATAGCGTAGCGTCTCGTGGGCTCGGAGATGT |
| Sequence-based reagent | PMID: 24097267:Ad2.10; Nextera barcode 710 | [PMID: 24097267](https://www.ncbi.nlm.nih.gov/pubmed/24097267) | library primers | CAAGCAGAAGACGGCATACGAGATCAGCCTCGGTCTCGTGGGCTCGGAGATGT |
| Sequence-based reagent | PMID: 24097267:Ad2.11; Nextera barcode 711 | [PMID: 24097267](https://www.ncbi.nlm.nih.gov/pubmed/24097267) | library primers | CAAGCAGAAGACGGCATACGAGATTGCCTCTTGTCTCGTGGGCTCGGAGATGT |
| Sequence-based reagent | PMID: 24097267:Ad2.12; Nextera barcode 712 | [PMID: 24097267](https://www.ncbi.nlm.nih.gov/pubmed/24097267) | library primers | CAAGCAGAAGACGGCATACGAGATTCCTCTACGTCTCGTGGGCTCGGAGATGT |
| Sequence-based reagent | PMID: 24097267:Ad1_noMX | [PMID: 24097267](https://www.ncbi.nlm.nih.gov/pubmed/24097267) | library primers | AATGATACGGCGACCACCGAGATCTACACTCGTCGGCAGCGTCAGATGTG |
| Sequence-based reagent | gfp_1F | this paper | RT-qpcr primers | ATGGCCAACACTTGTCACTA |
| Sequence-based reagent | gfp_1R | this paper | RT-qpcr primers | GGCATGGCACTCTTGAAAAA |
| Sequence-based reagent | pax6_1F | this paper | RT-qpcr primers | AGACGGACAGACTGGCAAAC |
| Sequence-based reagent | pax6_1R | this paper | RT-qpcr primers | TTCCATGTGCAAAAAGTCCA |
| Sequence-based reagent | actc_1F | this paper | RT-qpcr primers | GAGACCCTCTTCCAGCCTTC |
| Sequence-based reagent | actc_1R | this paper | RT-qpcr primers | GGTCCTTGCGGATATCAATG |
| Sequence-based reagent | tubb2b_1F | this paper | RT-qpcr primers | GCTGAAAGCAACATGAACGA |
| Sequence-based reagent | tubb2b_1R | this paper | RT-qpcr primers | CATCTTCCCCTTCCTCTTCC |
| Sequence-based reagent | odc1_1F | PMID: 23559567 | RT-qpcr primers, PMID: 23559567: odc1 | AGGCCACACTGGCAACTCA |
| Sequence-based reagent | odc1_1R | PMID: 23559567 | RT-qpcr primers, PMID: 23559567: odc1 | TGCGCTCAGTTCTGGTACTTCA |
| Sequence-based reagent | pbx3_ish_F | this paper | In situ hybridization primers | TGCAGGCAACTACAGTCCAG |
| Sequence-based reagent | pbx3_ish_R | this paper | In situ hybridization primers | TAATACGACTCACTATAGGGAGTAGTGATGTGCGGGTTGG |
| Sequence-based reagent | meis1_ish_F | this paper | In situ hybridization primers | ACGGGACTTCTGTGCATACC |
| Sequence-based reagent | meis1_ish_R | this paper | In situ hybridization primers | TAATACGACTCACTATAGGGGTGGCTGTTTTCTGCAATGA |
| Sequence-based reagent | l1cam_ish_F | this paper | In situ hybridization primers | CTCACTAGAGTGGACGGGGA |
| Sequence-based reagent | l1cam_ish_R | this paper | In situ hybridization primers | TAATACGACTCACTATAGGGAGTGCCTGACACAAGGACAG |
| Sequence-based reagent | uchl1_ish_F | this paper | In situ hybridization primers | AAATGTCCCTGGGGTGTTCC |
| Sequence-based reagent | uchl1_ish_R | this paper | In situ hybridization primers | TAATACGACTCACTATAGGGCACGGAGTTGTGAGCAGACT |
| Sequence-based reagent | nsg1_ish_F | this paper | In situ hybridization primers | GGGCATGGCTGAGATGACAA |
| Sequence-based reagent | nsg1_ish_R | this paper | In situ hybridization primers | TAATACGACTCACTATAGGGCGTAGGGCAAGGGGTAAGTG |
| Sequence-based reagent | ass1_ish_F | this paper | In situ hybridization primers | CCGGAATGTGAGTTTGTGCG |
| Sequence-based reagent | ass1_ish_R | this paper | In situ hybridization primers | TAATACGACTCACTATAGGGACTGCGCAAAGGGGTCTTTA |
| Sequence-based reagent | pbx3 MO1 | this paper | morpholino | CAAATCGTCGTACCTTTGAGCCATC |
| Sequence-based reagent | pbx3 MO2 | this paper | morpholino | TGACAACACAGACTGCAAGGAGACC |
| Sequence-based reagent | pbx3 MO2 VIVO | this paper | vivo-morpholino | TGACAACACAGACTGCAAGGAGACC |
| Sequence-based reagent | meis1 MO1 | this paper | morpholino | CATACTCTTCTCCGCAGCCTAATCA |
| Sequence-based reagent | meis1 MO2 | this paper | morpholino | AACTCCTTCCTACTTCAACTTCAGA |
| Sequence-based reagent | meis1 MO2 VIVO | this paper | vivo-morpholino | AACTCCTTCCTACTTCAACTTCAGA |
| Sequence-based reagent | pbx3 gRNA | this paper | sgRNA primers | CTAGCTAATACGACTCACTATAGGTGCCCGGCCCTTCTGTGGGTTTTAGAGCTAGAA |
| Sequence-based reagent | meis1 gRNA | this paper | sgRNA primers | CTAGCTAATACGACTCACTATAGGCATGGGGATCGCCATACAGTTTTAGAGCTAGAA |
| Sequence-based reagent | pbx3_hrm_1F | this paper | pcr primers | TCTCTTTCGAGTTAACGCATGA |
| Sequence-based reagent | pbx3_hrm_1R | this paper | pcr primers | GCACCCTCAGCTTGTAGAGAAT |
| Sequence-based reagent | meis1_hrm_1F | this paper | pcr primers | TTACACAGTACGACGATTTGCC |
| Sequence-based reagent | meis1_hrm_1R | this paper | pcr primers | TAAGGGAAGAAGTGACAGAGGG |
| Software, algorithm | Data analysis and figure production | this paper | source code and data analysis | <https://gitlab.com/akakebee/kakebeen-et-al-2019> |
