# Supplementary material for: Chromatin accessibility dynamics and single cell RNA-Seq reveal new regulators of regeneration in neural progenitors
Source: eLife. 2020 Apr 27;9:e52648. doi: 10.7554/eLife.52648 (PMC7250574; doi:10.7554/eLife.52648)
Supplement: Transparent reporting form [file elife-52648-transrepform.pdf]

eLife's transparent reporting form

We encourage authors to provide detailed information *within their submission* to facilitate the interpretation and replication of experiments. Authors can upload supporting documentation to indicate the use of appropriate reporting guidelines for health-related research (see EQUATOR Network), life science research (see the BioSharing Information Resource), or the ARRIVE guidelines for reporting work involving animal research. Where applicable, authors should refer to any relevant reporting standards documents in this form.

If you have any questions, please consult our Journal Policies and/or contact us: editorial@elifesciences.org.

Sample-size estimation

- You should state whether an appropriate sample size was computed when the study was being designed
- You should state the statistical method of sample size computation and any required assumptions
- If no explicit power analysis was used, you should describe how you decided what sample (replicate) size (number) to use

Please outline where this information can be found within the submission (e.g., sections or figure legends), or explain why this information doesn't apply to your submission:

No prior power analysis was calculated to determine the number of samples to sequence for ATAC-Seq per regeneration timepoint. Rather, sample sizes were determined based on best practices as reported by ENCODE (https://www.encodeproject.org/atac-seq/). Sample sizes for tail regeneration measurements were determined *a priori* based on previous analyses of the regenerating *Xenopus* tail.

Replicates

- You should report how often each experiment was performed
- You should include a definition of biological versus technical replication
- The data obtained should be provided and sufficient information should be provided to indicate the number of independent biological and/or technical replicates
- If you encountered any outliers, you should describe how these were handled
- Criteria for exclusion/inclusion of data should be clearly stated
- High-throughput sequence data should be uploaded before submission, with a private link for reviewers provided (these are available from both GEO and ArrayExpress)

Please outline where this information can be found within the submission (e.g., sections or figure legends), or explain why this information doesn't apply to your submission:

eLife Sciences Publications, Ltd is a limited liability non-profit non-stock corporation incorporated in the State of Delaware, USA, with company number 5030732, and is registered in the UK with company number FC030576 and branch number BR015634 at the address 1st Floor, 24 Hills Road, Cambridge CB2 1JP | August 2014

Here we define a biological replicate as a sample made from independent (or an independent set) of tadpoles whereas separate samples derived from the same individual tadpole (or set of individual tadpoles) would be considered technical replicates. ATAC-Seq libraries were made in biological triplicate for each tissue type and timepoint. After processing raw reads, samples were evaluate based on the number of reads and percent of reads aligned to the genome. ATAC-Seq libraries with less than one million aligned reads or with alignment rates less than 80% were not used for downstream analysis. These criteria restricted pax6_uninjured_replicate2 and pax6_0hpa_replicate1. Numbers of replicates, samples sizes, and numbers of cells used for library preparations are included in supplementary Table 1, the body of the text, and the Methods section. Morpholino/regeneration experiments were performed at least 2 times with different matings and used no less than 4 total tadpoles.

eLife Sciences Publications, Ltd is a limited liability non-profit non-stock corporation incorporated in the State of Delaware, USA, with company number 5030732, and is registered in the UK with company number FC030576 and branch number BR015634 at the address 1st Floor, 24 Hills Road, Cambridge CB2 1JP | August 2014

Statistical reporting

- Statistical analysis methods should be described and justified
- Raw data should be presented in figures whenever informative to do so (typically when N per group is less than 10)
- For each experiment, you should identify the statistical tests used, exact values of N, definitions of center, methods of multiple test correction, and dispersion and precision measures (e.g., mean, median, SD, SEM, confidence intervals; and, for the major substantive results, a measure of effect size (e.g., Pearson's r, Cohen's d)
- Report exact p-values wherever possible alongside the summary statistics and 95% confidence intervals. These should be reported for all key questions and not only when the p-value is less than 0.05.

Please outline where this information can be found within the submission (e.g., sections or figure legends), or explain why this information doesn't apply to your submission:

Statistical testing of qRT-PCR and tail regeneration measurements used parametric tests. The test type and significance values calculated can be found in figure legends of Supplementary Figure 1 (qRT-PCR) and Figure 6 (tail measurements). Single cell RNA-Seq used statistical tests designed specifically for such analyses. ATAC-Seq used statistical tests from the R package edgeR.

(For large datasets, or papers with a very large number of statistical tests, you may upload a single table file with tests, Ns, etc., with reference to sections in the manuscript.)

Group allocation

- Indicate how samples were allocated into experimental groups (in the case of clinical studies, please specify allocation to treatment method); if randomization was used, please also state if restricted randomization was applied
- Indicate if masking was used during group allocation, data collection and/or data analysis

Please outline where this information can be found within the submission (e.g., sections or figure legends), or explain why this information doesn't apply to your submission:

Individual tadpoles were allocated randomly into groups.

Additional data files ("source data")

- We encourage you to upload relevant additional data files, such as numerical data that are represented as a graph in a figure, or as a summary table
- Where provided, these should be in the most useful format, and they can be uploaded as "Source data" files linked to a main figure or table
- Include model definition files including the full list of parameters used
- Include code used for data analysis (e.g., R, MatLab)
- Avoid stating that data files are "available upon request"

Please indicate the figures or tables for which source data files have been provided:

eLife Sciences Publications, Ltd is a limited liability non-profit non-stock corporation incorporated in the State of Delaware, USA, with company number 5030732, and is registered in the UK with company number FC030576 and branch number BR015634 at the address 1st Floor, 24 Hills Road, Cambridge CB2 1JP | August 2014

Steps taken to process raw data of ATAC-Seq and scRNA-Seq are supplied in the Methods section. Data relevant to analyses are included in supplementary Tables and code and source files for making figures from sequencing data in R are available on GitLab (https://gitlab.com/akakebee/kakebeen-et-al-2019). Raw ATAC-Seq and scRNA-Seq data will be available on GEO (accession number to be assigned). Other data are displayed already in figures.

eLife Sciences Publications, Ltd is a limited liability non-profit non-stock corporation incorporated in the State of Delaware, USA, with company number 5030732, and is registered in the UK with company number FC030576 and branch number BR015634 at the address 1st Floor, 24 Hills Road, Cambridge CB2 1JP | August 2014
